# The genome sequence of the jumping weevil, *Orchestes rusci *(Herbst, 1795)

**DOI:** 10.12688/wellcomeopenres.22745.1

**Published:** 2024-07-22

**Authors:** Stephen Moran, Natural History Museum Genome Acquisition Lab, Darwin Tree of Life Barcoding collective, Wellcome Sanger Institute Tree of Life Management, Samples and Laboratory team, Wellcome Sanger Institute Scientific Operations: Sequencing Operations, Wellcome Sanger Institute Tree of Life Core Informatics team, Tree of Life Core Informatics collective, Darwin Tree of Life Consortium

**Affiliations:** 1 Highland Biological Recording Group, Inverness, Scotland, UK

**Keywords:** Orchestes rusci, jumping weevil, genome sequence, chromosomal, Coleoptera

## Abstract

We present a genome assembly from an individual female *Orchestes rusci* (the jumping weevil; Arthropoda; Insecta; Coleoptera; Curculionidae). The genome sequence spans 624.00 megabases. Most of the assembly is scaffolded into 12 chromosomal pseudomolecules, including the X sex chromosome. The mitochondrial genome has also been assembled and is 21.73 kilobases in length.

## Species taxonomy

Eukaryota; Opisthokonta; Metazoa; Eumetazoa; Bilateria; Protostomia; Ecdysozoa; Panarthropoda; Arthropoda; Mandibulata; Pancrustacea; Hexapoda; Insecta; Dicondylia; Pterygota; Neoptera; Endopterygota; Coleoptera; Polyphaga; Cucujiformia; Curculionoidea; Curculionidae; Curculioninae; Rhamphini; Rhamphina; *Orchestes*: *Orchestes* ( *Alyctus*) *rusci* (J.F.W. Herbst, 1795)(NCBI:txid878341).

## Background

The jumping weevil, or flea weevil, *Orchestes rusci* (J.F.W. Herbst, 1795) is one of nine species in the genus which were previously included in *Rhynchaenus* Clairville. *O. rusci*, along with two other UK species has been assigned to the subgenus *Alyctus* Thomson, CG ( Duff, 2016). In common with most other weevils of the tribe Rhamphini, it possesses strongly developed hind femora adapted for jumping. It can be separated from the other eight *Orchestes* species by the absence of sharp ventral tubercles on the hind femur.

**Figure 1.**
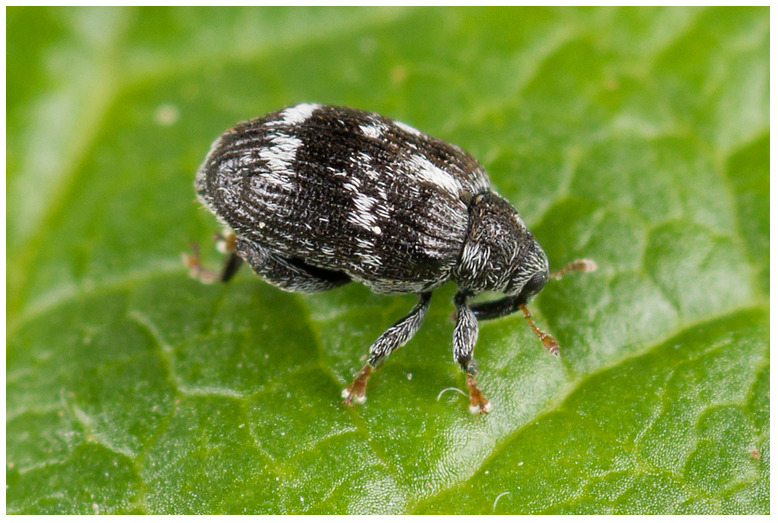
Photograph of *Orchestes rusci* (not the specimen used for genome sequencing) by Nikolai Vladimirov.

Ranging from 2.2 to 2.5 mm in length and roughly oval in shape, *O. rusci* has a black body with yellowish antennae and tarsi. The upper side is sparsely covered in a white pubescence, slightly thicker along the base of the elytral suture and also across the elytrae where they form two irregular but distinct white bands ( Figure 1).

*Orchestes rusci* occurs in mixed deciduous woodland and the drier parts of fens, bogs and heaths where the host plants occur. It is found on birch, probably on both *Betula pendula* Roth. and *B pubescens* Ehrh. and on their hybrid, in the UK. The adults overwinter and are active from May to September, peaking in June and July. The species is thought to be univoltine in northern areas ( Duff, 2016; Morris, 2012; UK Beetles, 2024). Known from most of the Palearctic region from the Pyrenees north to the UK and Fennoscandia and from Ireland east to Russia and Japan ( GBIF Secretariat, 2024).

The females chew into the apical margins of leaves and usually lay a single egg in early to mid-summer. Starting at the tip, the larvae mine the edge of the leaf until about the middle when they turn towards the centre producing a mine that widens along its length. The mine terminates in an almost circular blotch against the midrib in which the larva pupates, having stitched the upper and lower epidermis together. This disc is generally excised and falls to the ground. The new adults emerge from July onwards, overlapping with the previous generation but not reproducing until the following year ( UK Beetles, 2024).

## Genome sequence report

The genome of an adult female *Orchestes rusci* was sequenced using Pacific Biosciences single-molecule HiFi long reads, generating a total of 22.47 Gb (gigabases) from 2.67 million reads, providing approximately 34-fold coverage. Primary assembly contigs were scaffolded with chromosome conformation Hi-C data, which produced 114.65 Gbp from 759.25 million reads, yielding an approximate coverage of 184-fold. Specimen and sequencing information is summarised in Table 1.

**Table 1.** Specimen and sequencing data for *Orchestes rusci*.

Project information			
**Study title**	Orchestes rusci		
**Umbrella BioProject**	PRJEB62726		
**Species**	*Orchestes rusci*		
**BioSample**	SAMEA110029153		
**NCBI taxonomy ID**	878341		
Specimen information			
**Technology**	**ToLID**	**BioSample accession**	**Organism part**
**PacBio long read sequencing**	icOrcRusc1	SAMEA14448475	Whole organism
**Hi-C sequencing**	icOrcRusc1	SAMEA14448475	Whole organism
Sequencing information			
**Platform**	**Run accession**	**Read count**	**Base count (Gb)**
**Hi-C Illumina NovaSeq 6000**	ERR11526203	7.59e+08	114.65
**PacBio Sequel IIe**	ERR11512311	2.67e+06	22.47

**Figure 2.**
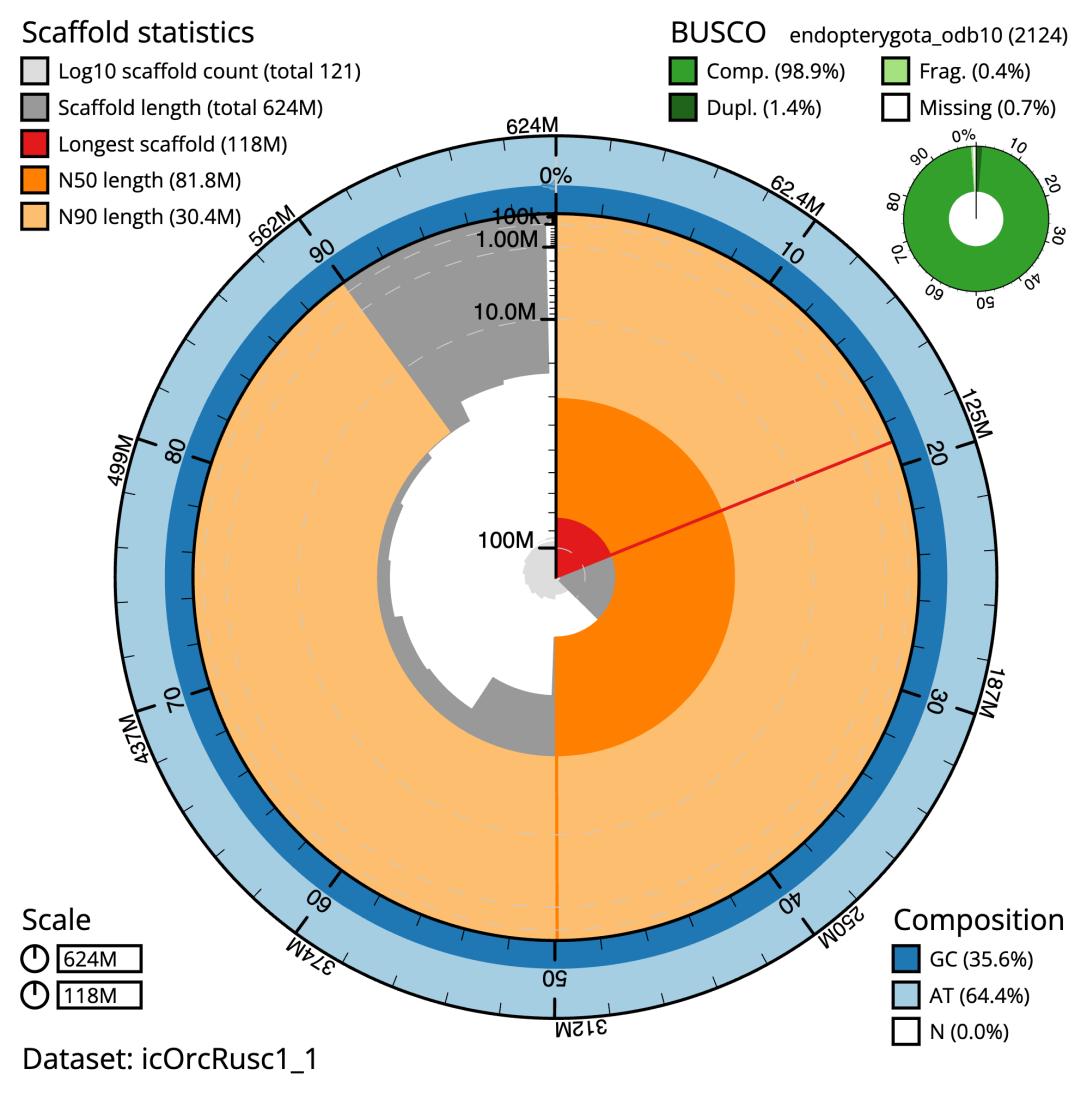
Genome assembly of *Orchestes rusci*, icOrcRusc1.1: metrics. The BlobToolKit snail plot shows N50 metrics and BUSCO gene completeness. The main plot is divided into 1,000 size-ordered bins around the circumference with each bin representing 0.1% of the 624,046,445 bp assembly. The distribution of scaffold lengths is shown in dark grey with the plot radius scaled to the longest scaffold present in the assembly (118,147,993 bp, shown in red). Orange and pale-orange arcs show the N50 and N90 scaffold lengths (81,830,918 and 30,363,857 bp), respectively. The pale grey spiral shows the cumulative scaffold count on a log scale with white scale lines showing successive orders of magnitude. The blue and pale-blue area around the outside of the plot shows the distribution of GC, AT and N percentages in the same bins as the inner plot. A summary of complete, fragmented, duplicated and missing BUSCO genes in the endopterygota_odb10 set is shown in the top right. An interactive version of this figure is available at https://blobtoolkit.genomehubs.org/view/Orchestes%20rusci/dataset/icOrcRusc1_1/snail.

**Figure 3.**
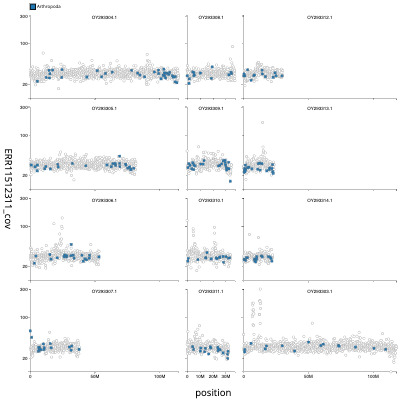
Genome assembly of *Orchestes rusci*, icOrcRusc1.1: Distribution plot of base coverage in ERR11512311 against position for sequences in assembly icOrcRusc1_1. Windows of 100 kb are coloured by phylum. The assembly has been filtered to exclude sequences with length < 2,550,000. An interactive version of this figure may be viewed here.

**Figure 4.**
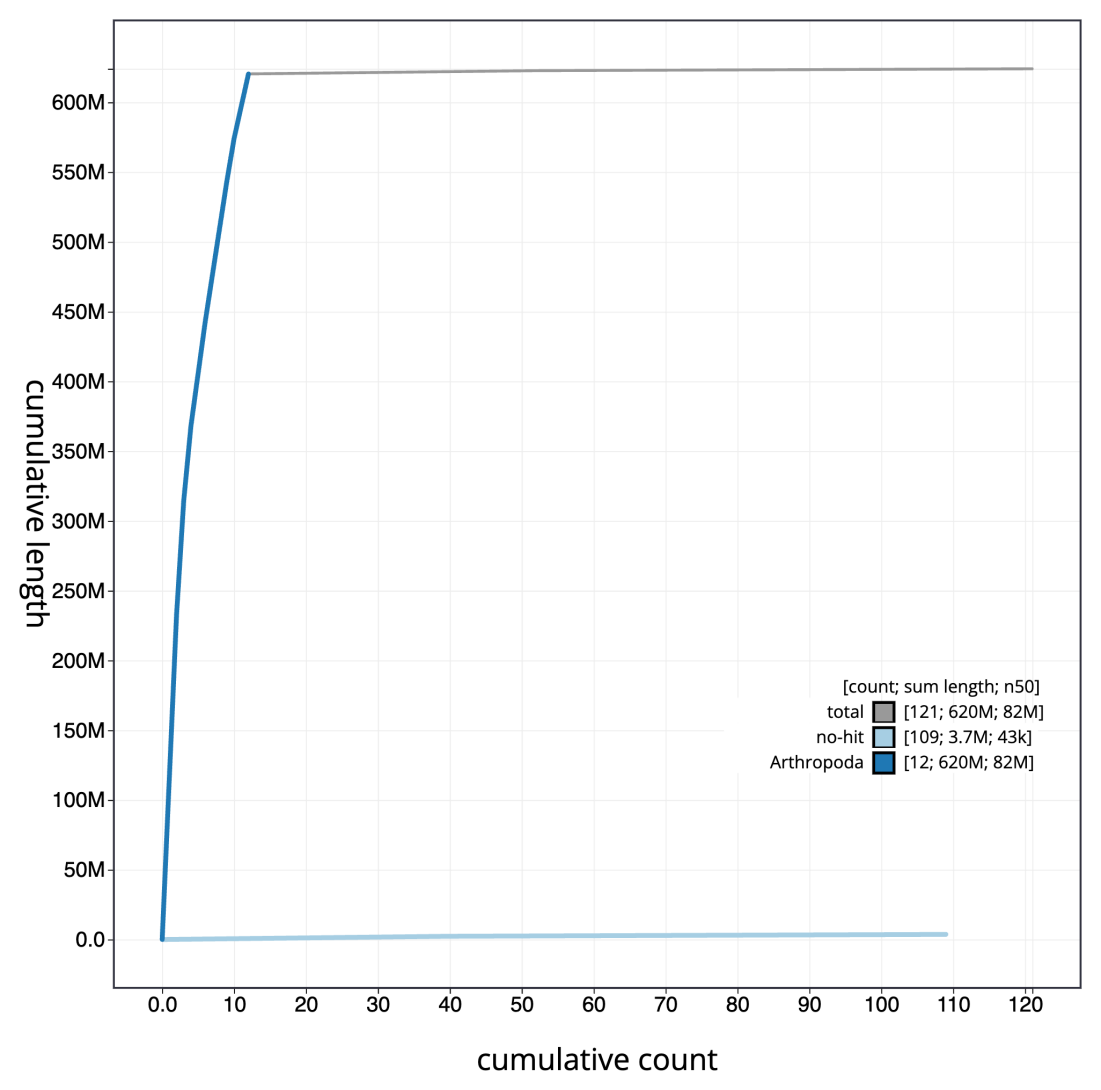
Genome assembly of *Orchestes rusci* icOrcRusc1.1: BlobToolKit cumulative sequence plot. The grey line shows cumulative length for all sequences. Coloured lines show cumulative lengths of sequences assigned to each phylum using the buscogenes taxrule. An interactive version of this figure is available at https://blobtoolkit.genomehubs.org/view/Orchestes%20rusci/dataset/icOrcRusc1_1/cumulative.

**Figure 5.**
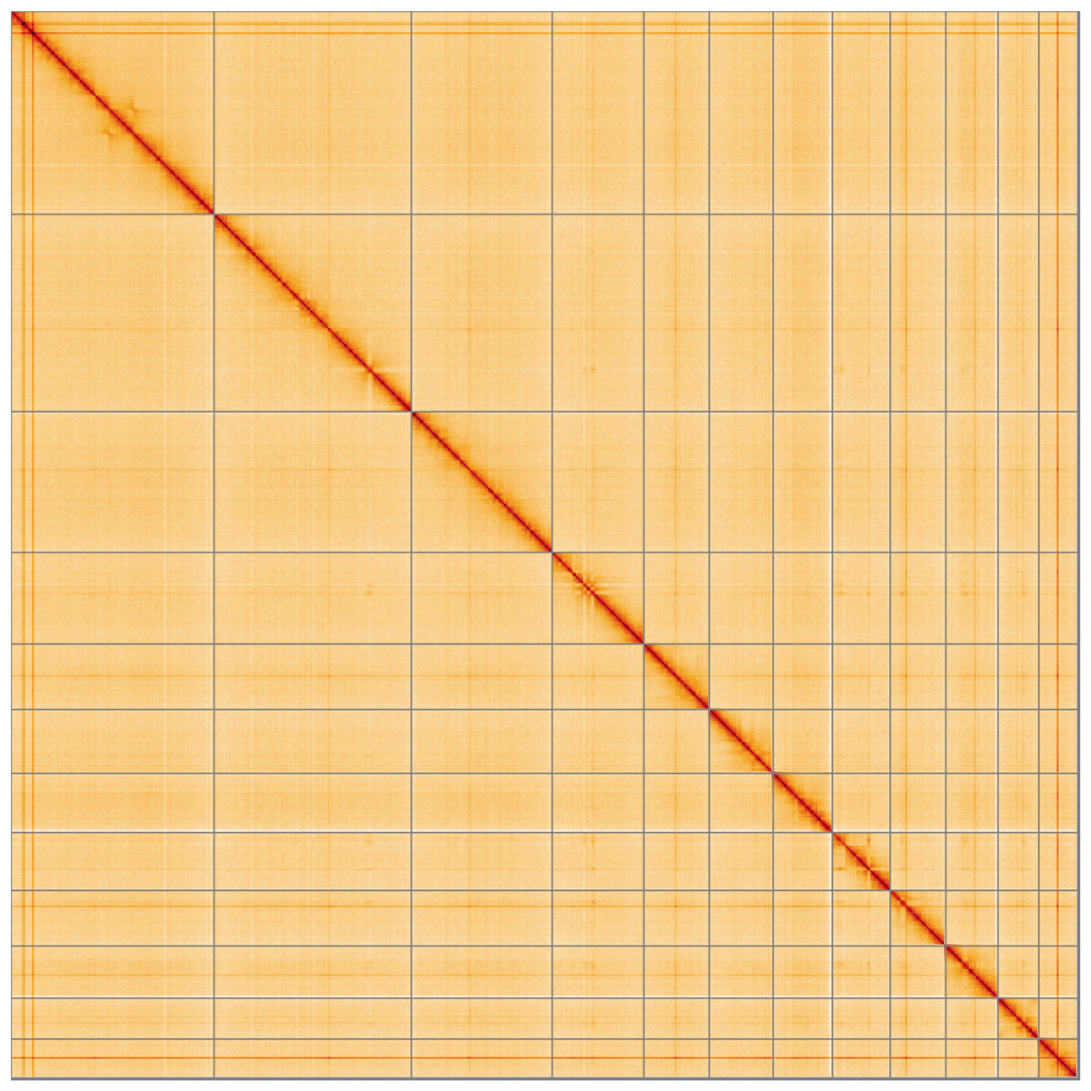
Genome assembly of *Orchestes rusci* icOrcRusc1.1: Hi-C contact map of the icOrcRusc1.1 assembly, visualised using HiGlass. Chromosomes are shown in order of size from left to right and top to bottom. An interactive version of this figure may be viewed at https://genome-note-higlass.tol.sanger.ac.uk/l/?d=TsqTbYaDSSmpj65xkMw_3w.

Manual assembly curation corrected 64 missing joins or mis-joins and 11 haplotypic duplications, reducing the assembly length by 0.43% and the scaffold number by 15.38%, and increasing the scaffold N50 by 56.44%. The final assembly has a total length of 624.00 Mb in 120 sequence scaffolds with a scaffold N50 of 81.8 Mb ( Table 2). The total count of gaps in the scaffolds is 812. The snail plot in Figure 2 provides a summary of the assembly statistics, while Figure 3 shows the distribution of assembly scaffolds based on base coverage across chromosomes. The cumulative assembly plot in Figure 4 shows curves for subsets of scaffolds assigned to different phyla. Most (99.42%) of the assembly sequence was assigned to 12 chromosomal-level scaffolds, representing 11 autosomes and the X sex chromosome. Chromosome-scale scaffolds confirmed by the Hi-C data are named in order of size ( Figure 5; Table 3). Chromosome X was assigned based on synteny to *Philonthus cognatus* (GCA_932526585.2) ( Crowley *et al.*, 2023b) and *Cetonia aurata* (GCA_949128085.1) ( Grayson *et al.*, 2023). While not fully phased, the assembly deposited is of one haplotype. Contigs corresponding to the second haplotype have also been deposited. The mitochondrial genome was also assembled and can be found as a contig within the multifasta file of the genome submission.

**Table 2.** Genome assembly data for *Orchestes rusci*, icOrcRusc1.1.

Genome assembly		
Assembly name	icOrcRusc1.1	
Assembly accession	GCA_958502075.1	
*Accession of alternate haplotype*	*GCA_958502085.1*	
Span (Mb)	624.00	
Number of contigs	933	
Contig N50 length (Mb)	1.2	
Number of scaffolds	120	
Scaffold N50 length (Mb)	81.8	
Longest scaffold (Mb)	118.15	
Assembly metrics *		*Benchmark*
Consensus quality (QV)	60.7	*≥ 50*
*k*-mer completeness	100.0%	*≥ 95%*
BUSCO **	C:98.9%[S:97.5%,D:1.4%],F:0.4%,M:0.7%,n:2,124	*C ≥ 95%*
Percentage of assembly mapped to chromosomes	99.42%	*≥ 95%*
Sex chromosomes	X	*localised homologous pairs*
Organelles	Mitochondrial genome: 21.73 kb	*complete single alleles*

* Assembly metric benchmarks are adapted from column VGP-2020 of “Table 1: Proposed standards and metrics for defining genome assembly quality” from Rhie *et al.* (2021). ** BUSCO scores based on the endopterygota_odb10 BUSCO set using version 5.3.2. C = complete [S = single copy, D = duplicated], F = fragmented, M = missing, n = number of orthologues in comparison. A full set of BUSCO scores is available at https://blobtoolkit.genomehubs.org/view/Orchestes_rusci/dataset/GCA_958502075.1/busco.

**Table 3.** Chromosomal pseudomolecules in the genome assembly of *Orchestes rusci*, icOrcRusc1.

INSDC accession	Name	Length (Mb)	GC%
OY293304.1	1	114.73	35.5
OY293305.1	2	81.83	35.5
OY293306.1	3	53.39	35.5
OY293307.1	4	38.04	35.5
OY293308.1	5	37.14	35.5
OY293309.1	6	34.41	35.5
OY293310.1	7	33.61	35.5
OY293311.1	8	32.4	35.5
OY293312.1	9	30.36	35.5
OY293313.1	10	23.76	35.5
OY293314.1	11	22.55	35.5
OY293303.1	X	118.15	35.5
OY293315.1	MT	0.02	32.0

The estimated Quality Value (QV) of the final assembly is 60.7 with *k*-mer completeness of 100.0%, and the assembly has a BUSCO v5.3.2 completeness of 98.9% (single = 97.5%, duplicated = 1.4%), using the endopterygota_odb10 reference set ( *n* = 2,124).

Metadata for specimens, BOLD barcode results, spectra estimates, sequencing runs, contaminants and pre-curation assembly statistics are given at https://links.tol.sanger.ac.uk/species/878341.

## Methods

### Sample acquisition

An adult female *Orchestes rusci* (specimen ID NHMUK014451660, ToLID icOrcRusc1) was collected from Beinn Eighe National Nature Reserve, Scotland, UK (latitude 57.63, longitude –5.35) on 2021-09-09. The specimen was collected and identified by Stephen Moran (Highland Biological Recording Group) and preserved by dry frozen at –80 °C.

In addition to identification based on morphology, the species taxonomy was verified by DNA barcoding soon after collection, according to the framework developed by Twyford *et al*. (2024). A small sample was dissected from the specimen and stored in ethanol. The tissue was lysed, and the COI marker region was amplified by PCR. Amplicons were sequenced and compared to the BOLD database, confirming the species identification ( Crowley *et al.*, 2023a). The standard operating procedures for the Darwin Tree of Life barcoding have been deposited on protocols.io ( Beasley *et al.*, 2023). The remaining parts of the specimen were shipped on dry ice to the Wellcome Sanger Institute (WSI). A DNA barcode was also generated from the PacBio sequencing data at a later stage for sample tracking through the genome production pipeline at the WSI ( Twyford *et al.*, 2024).

### Nucleic acid extraction

The workflow for high molecular weight (HMW) DNA extraction at the Wellcome Sanger Institute (WSI) Tree of Life Core Laboratory includes a sequence of core procedures: sample preparation; sample homogenisation, DNA extraction, fragmentation, and clean-up. In sample preparation, the icOrcRusc1 sample was weighed and dissected on dry ice ( Jay *et al.*, 2023). Tissue from whole organism was homogenised using a PowerMasher II tissue disruptor ( Denton *et al.*, 2023a).

HMW DNA was extracted in the WSI Scientific Operations core using the Automated MagAttract v2 protocol ( Oatley *et al.*, 2023). The DNA was sheared into an average fragment size of 12–20 kb in a Megaruptor 3 system with speed setting 31 ( Bates *et al.*, 2023). Sheared DNA was purified by solid-phase reversible immobilisation ( Strickland *et al.*, 2023): in brief, the method employs a 1.8X ratio of AMPure PB beads to sample to eliminate shorter fragments and concentrate the DNA. The concentration of the sheared and purified DNA was assessed using a Nanodrop spectrophotometer and Qubit Fluorometer using the Qubit dsDNA High Sensitivity Assay kit. Fragment size distribution was evaluated by running the sample on the FemtoPulse system.

Protocols developed by the WSI Tree of Life laboratory are publicly available on protocols.io ( Denton *et al.*, 2023b).

### Sequencing

Pacific Biosciences HiFi circular consensus DNA sequencing libraries were constructed according to the manufacturers’ instructions. DNA sequencing was performed by the Scientific Operations core at the WSI on a Pacific Biosciences Sequel IIe instrument. Hi-C data were also generated from whole organism tissue of icOrcRusc1 using the Arima-HiC v2 kit. The Hi-C sequencing was performed using paired-end sequencing with a read length of 150 bp on the Illumina NovaSeq 6000 instrument.

### Genome assembly, curation and evaluation

***Assembly***

The original assembly of HiFi reads was performed using Hifiasm ( Cheng *et al.*, 2021) with the --primary option. Haplotypic duplications were identified and removed with purge_dups ( Guan *et al.*, 2020). Hi-C reads are further mapped with bwa-mem2 ( Vasimuddin *et al.*, 2019) to the primary contigs, which are further scaffolded using the provided Hi-C data ( Rao *et al.*, 2014) in YaHS ( Zhou *et al.*, 2023) using the --break option. Scaffolded assemblies are evaluated using Gfastats ( Formenti *et al.*, 2022), BUSCO ( Manni *et al.*, 2021) and MERQURY.FK ( Rhie *et al.*, 2020).

The mitochondrial genome was assembled using MitoHiFi ( Uliano-Silva *et al.*, 2023), which runs MitoFinder ( Allio *et al.*, 2020) and uses these annotations to select the final mitochondrial contig and to ensure the general quality of the sequence.

***Assembly curation***

The assembly was decontaminated using the Assembly Screen for Cobionts and Contaminants (ASCC) pipeline (article in preparation). Flat files and maps used in curation were generated in TreeVal ( Pointon *et al.*, 2023). Manual curation was primarily conducted using PretextView ( Harry, 2022), with additional insights provided by JBrowse2 ( Diesh *et al.*, 2023) and HiGlass ( Kerpedjiev *et al.*, 2018). Scaffolds were visually inspected and corrected as described by Howe *et al*. (2021). Any identified contamination, missed joins, and mis-joins were corrected, and duplicate sequences were tagged and removed. he entire process is documented at https://gitlab.com/wtsi-grit/rapid-curation (article in preparation).

***Evaluation of the final assembly***

A Hi-C map for the final assembly was produced using bwa-mem2 ( Vasimuddin *et al.*, 2019) in the Cooler file format ( Abdennur & Mirny, 2020). To assess the assembly metrics, the *k*-mer completeness and QV consensus quality values were calculated in Merqury ( Rhie *et al.*, 2020). This work was done using Nextflow ( Di Tommaso *et al.*, 2017) DSL2 pipelines “sanger-tol/readmapping” ( Surana *et al.*, 2023a) and “sanger-tol/genomenote” ( Surana *et al.*, 2023b). The genome was analysed within the BlobToolKit environment ( Challis *et al.*, 2020) and BUSCO scores ( Manni *et al.*, 2021; Simão *et al.*, 2015) were calculated.

The genome assembly and evaluation pipelines were developed using the nf-core tooling ( Ewels *et al.*, 2020), use MultiQC ( Ewels *et al.*, 2016), and make extensive use of the Conda package manager, the Bioconda initiative ( Grüning *et al.*, 2018), the Biocontainers infrastructure ( da Veiga Leprevost *et al.*, 2017), and the Docker ( Merkel, 2014) and Singularity ( Kurtzer *et al.*, 2017) containerisation solutions.

Table 4 contains a list of relevant software tool versions and sources.

**Table 4.** Software tools: versions and sources.

Software tool	Version	Source
BlobToolKit	4.2.1	https://github.com/blobtoolkit/blobtoolkit
BUSCO	5.3.2	https://gitlab.com/ezlab/busco
Hifiasm	0.16.1-r375	https://github.com/chhylp123/hifiasm
HiGlass	1.11.6	https://github.com/higlass/higlass
Merqury	MerquryFK	https://github.com/thegenemyers/MERQURY.FK
MitoHiFi	3	https://github.com/marcelauliano/MitoHiFi
PretextView	0.2	https://github.com/sanger-tol/PretextView
purge_dups	1.2.5	https://github.com/dfguan/purge_dups
sanger-tol/genomenote	v1.0	https://github.com/sanger-tol/genomenote
sanger-tol/readmapping	1.1.0	https://github.com/sanger-tol/readmapping/tree/1.1.0
YaHS	1.2a.2	https://github.com/c-zhou/yahs

### Wellcome Sanger Institute – Legal and Governance

The materials that have contributed to this genome note have been supplied by a Darwin Tree of Life Partner. The submission of materials by a Darwin Tree of Life Partner is subject to the **‘Darwin Tree of Life Project Sampling Code of Practice’**, which can be found in full on the Darwin Tree of Life website here. By agreeing with and signing up to the Sampling Code of Practice, the Darwin Tree of Life Partner agrees they will meet the legal and ethical requirements and standards set out within this document in respect of all samples acquired for, and supplied to, the Darwin Tree of Life Project.

Further, the Wellcome Sanger Institute employs a process whereby due diligence is carried out proportionate to the nature of the materials themselves, and the circumstances under which they have been/are to be collected and provided for use. The purpose of this is to address and mitigate any potential legal and/or ethical implications of receipt and use of the materials as part of the research project, and to ensure that in doing so we align with best practice wherever possible. The overarching areas of consideration are: 

•   Ethical review of provenance and sourcing of the material

•   Legality of collection, transfer and use (national and international)

Each transfer of samples is further undertaken according to a Research Collaboration Agreement or Material Transfer Agreement entered into by the Darwin Tree of Life Partner, Genome Research Limited (operating as the Wellcome Sanger Institute), and in some circumstances other Darwin Tree of Life collaborators.

## Data Availability

European Nucleotide Archive: *Orchestes rusci*. Accession number PRJEB62726; https://identifiers.org/ena.embl/PRJEB62726 ( Wellcome Sanger Institute, 2023). The genome sequence is released openly for reuse. The *Orchestes rusci* genome sequencing initiative is part of the Darwin Tree of Life (DToL) project. All raw sequence data and the assembly have been deposited in INSDC databases. The genome will be annotated using available RNA-Seq data and presented through the Ensembl pipeline at the European Bioinformatics Institute. Raw data and assembly accession identifiers are reported in Table 1 and Table 2.

## Author information

Members of the Natural History Museum Genome Acquisition Lab are listed here: https://doi.org/10.5281/zenodo.12159242.

Members of the Darwin Tree of Life Barcoding collective are listed here: https://doi.org/10.5281/zenodo.12158331

Members of the Wellcome Sanger Institute Tree of Life Management, Samples and Laboratory team are listed here: https://doi.org/10.5281/zenodo.12162482.

Members of Wellcome Sanger Institute Scientific Operations: Sequencing Operations are listed here: https://doi.org/10.5281/zenodo.12165051.

Members of the Wellcome Sanger Institute Tree of Life Core Informatics team are listed here: https://doi.org/10.5281/zenodo.12160324.

Members of the Tree of Life Core Informatics collective are listed here: https://doi.org/10.5281/zenodo.12205391.

Members of the Darwin Tree of Life Consortium are listed here: https://doi.org/10.5281/zenodo.4783558.
