# Supplementary material for: The genome sequence of the jumping weevil, Orchestes rusci (Herbst, 1795)
Source: Wellcome Open Res. 2024 Jul 22;9:398. [Version 1] doi: 10.12688/wellcomeopenres.22745.1 (PMC11406132; doi:10.12688/wellcomeopenres.22745.1)
Supplement: Peer review history

# Reviewer report: Reviewer response for version 1

**DOI:** 10.21956/wellcomeopenres.25048.r98652

**Published:** 2024-09-16

**Authors:** Gözde Güney

**Affiliations:** 1 University of Göttingen, Göttingen, Germany

Data noted by the author reports a high quality of genome assembly from *Orchestes rusci. *The method is appropriate for the assembly, the author provides sufficient quality control, and the assembly is publicly available. 

**Point 1:** "... usually lay a single egg in early to mid-summer." I believe laying a single egg means per leaf, which is unclear in the text, please check and correct if necessary.

Are sufficient details of methods and materials provided to allow replication by others?

Yes

Is the rationale for creating the dataset(s) clearly described?

Yes

Are the datasets clearly presented in a useable and accessible format?

Yes

Are the protocols appropriate and is the work technically sound?

Yes

Reviewer Expertise:

I work on beetle pests using omics methods.

I confirm that I have read this submission and believe that I have an appropriate level of expertise to confirm that it is of an acceptable scientific standard.

# Reviewer report: Reviewer response for version 1

**DOI:** 10.21956/wellcomeopenres.25048.r94472

**Published:** 2024-09-05

**Authors:** Lindsey Perkin

**Affiliations:** 1 USDA-ARS Southern Plains Agricultural Research Center (Ringgold ID: 57708), College Station, Texas, USA

The manuscript reports on a reference genome for *Orchestes rusci*, the jumping weevil. This paper contributes a Curculionidae genome to the database- a group that is very specious, with few representatives sequenced. The authors do a good job listing out their methods and results. The only part that was a little difficult to follow was the background section on which genus the jumping weevil is classified.

Are sufficient details of methods and materials provided to allow replication by others?

Yes

Is the rationale for creating the dataset(s) clearly described?

Yes

Are the datasets clearly presented in a useable and accessible format?

Yes

Are the protocols appropriate and is the work technically sound?

Yes

Reviewer Expertise:

genomics, transcriptomics, Curculioninae

I confirm that I have read this submission and believe that I have an appropriate level of expertise to confirm that it is of an acceptable scientific standard.
